# Spent *Pleurotus* substrate as organic fertilizer to improve yield and soil fertility: the case of baby leaf lettuce production

**DOI:** 10.1002/jsfa.14303

**Published:** 2025-05-28

**Authors:** Marina De Bonis, Paolo Sambo, Giampaolo Zanin, Mariateresa Cardarelli, Carlo Nicoletto

**Affiliations:** ^1^ Department of Agronomy, Food, Natural resources, Animals and Environment (DAFNAE) University of Padova Legnaro Italy; ^2^ Department of Agricultural and Forestry Sciences (DAFNE) University of Tuscia Viterbo Italy

**Keywords:** spent mushrooms substrate, organic fertilization, greenhouse tunnel, soil quality, nitrogen indices, vegetables, circularity

## Abstract

**BACKGROUND:**

Spent mushrooms substrate is the most important waste generated by edible mushrooms industry and can be re‐used as organic fertilizer, following sustainable production principles. In the present study, Spent *Pleurotus* substrate (SPS) was used for organic fertilization of baby leaf production during two consecutive cycles (1st: October to December; 2nd: December to March) using two variety of lettuce (Doge and Imperiale). Different amounts of SPS in a mix with chemical fertilizers were applied: 100% SPS to fulfil the N needs of the crop and 50% of mineral N (T100 + 50), 200% SPS to fulfil the double N needs (T200) and 200% SPS to fulfil the N needs of the crop and 50% of mineral N (T200 + 50). SPS treatments were compared with an unfertilized control (T0) and chemical fertilization treatments. The crops coverage, Soil Plant Analysis Development index (i.e. SPAD) and yield were monitored during each cycle and soil samples were analysed to observe the effect of SPS on soil fertility.

**RESULTS:**

Among SPS treatments, T100 + 50, which combined 50% mineral fertilization with SPS, yielded the best productive results among SPS treatments. This combination improved nutrient availability, whereas higher SPS concentrations (T200, T200 + 50) reduced yields due to nitrogen immobilization. The high initial soil nitrogen content limited significant changes in soil nitrogen, but SPS improved soil organic matter, active carbon and enzyme activity, enhancing microbial activity. Among the two lettuce varieties a difference in nitrogen uptake and utilization was observed.

**CONCLUSION:**

Overall, the study highlights the potential of raw SPS as a sustainable organic fertilizer for baby leaf cultivation. Incorporating SPS alongside half of the crop's mineral nitrogen requirement offers a promising alternative to conventional mineral fertilization. After just two crop cycles, this approach maintains a good yield at the same time as enhancing soil organic matter and enzymatic activity, ultimately improving soil fertility. © 2025 The Author(s). *Journal of the Science of Food and Agriculture* published by John Wiley & Sons Ltd on behalf of Society of Chemical Industry.

## INTRODUCTION

The world mushroom industry production is expanding in the last years. In 2021, FAOSTAT calculated that 44 million tons of mushrooms and truffles were produced worldwide.^1^ Singh *et al*.^2^ estimated that industrial production of edible mushrooms could reach 50 million tons in 2025 looking at the growing patterns of the major productive countries. The most important waste generated by this industry is the spent mushroom substrate (SMS), which is the cultivation substrate at the end of the production cycle. It is mostly composed by degraded lignocellulosic materials made of agricultural bioproducts, residual fungal mycelium, nutrients and enzymes. It has been calculated that, on average, 5 kg of spent mushrooms substrate is produced for every kg of edible mushrooms.^3^ This material is usually discarded after the production and, for the industry, the amount of waste generated is hard to be managed.^4^ The composition of SMS can be variable according to the cultivated mushrooms species produced. Nowadays, most literature refers to SMS derived from *Agaricus bisporus* cultivation, which is the most important edible mushroom produced in the western regions. The SMS of *Agaricus bisporus* is generally composed by wheat straw, horse and chicken manure and it is generally characterized by a C/N ratio of 15.^5^ The second most important cultivated mushroom in Europe is *Pleurotus* spp. and the typical cultivation substrate is composed of straw or different agro‐waste residues (pasteurized or sterilized) and different forms of nitrogen supplementation to reach a C/N ratio around 40‐50 at the end of the cycle.^6^

According to the circular economy theory, at the end of this production chain, these wastes should be used as resource for other purposes. In recent decades, many studies have been carried out on the reuse of SMS such as for bioremediation,^4^, ^5^, ^6^, ^7^ production of energy through biogas facilities,^8^, ^9^ alternative feed for animal rearing,^10^ production of others edible mushrooms,^11^ cultivation substrate for nursery plant production^12^, ^13^ and soil organic fertilization.^10^, ^14^, ^15^ The application of SMS as organic fertilizer has been the focus of extensive research in recent years, with experiments involving different vegetables.^5^, ^13^, ^16^, ^17^, ^18^, ^19^ SMS application can have positive effects on soil quality, including reducing soil‐borne disease pathogens,^20^ decreasing bulk density, and enhancing soil aggregation and organic matter content.^21^ Chen *et al*.^14^ showed that SMS from different mushroom productions can be adopted as organic fertilization and can promote nitrogen, phosphorus and potassium in an open field rotation of corn and wheat. Most of the literature regarding organic fertilization observed the effect of spent *Pleurotus* substrate (SPS) in a mixture with spent *Agaricus* substrate or after a post‐processing treatment such as composting.^21^ A few studies have been conducted using only raw SMS from *Pleurotus* cultivation, such as that by Somnath *et al*.^18^ who cultivated *Capsicum annum* in pots. However, raw SPS has never been used in a trial with leafy vegetables. The application of SPS as fertilizer could decrease the environmental impact of intensive cropping systems as leafy vegetables.

In particular, baby leaf lettuce cultivations are extremely intensive with an average of six growing cycles and almost 10 cuts per year. The duration of each cycle can range between 25 and 30 days in the spring–summer, up to 60 days during the winter season.^22^ The high number of crop cycles leads to intense soil tillage, high chemical fertilization input and faster mineralization of soil organic matter.^23^ Fertilization is a key factor for baby leaf production because the nitrate content in the commercial product must adhere to Reg. UE 2023/915.^24^ Furthermore, chemical fertilization in continuous cropping succession and short cycles can be an environmental issue as a result of the soil nitrogen leaching and its consequential negative impacts on ecosystem functioning.^25^, ^26^ Therefore, using a sustainable source of nutrient and organic matter for baby leaf production is essential.^23^

For this reason, the the present study aimed to evaluate whether the application of raw SPS is suitable for the production of baby leaf lettuce in a protected environment, for which, with respect to its productive characteristics, there is a need to find an alternative method of maintaining crop yield and enhancing soil fertility. During a trial lasting 6 months, the effect of application of different amount of SPS was observed in depth via measurements on crop production, N use efficiency and soil fertility with a particular focus on enzyme activities.

## MATERIALS AND METHODS

### Experiment set‐up

The experiment took place in a multispan plastic greenhouse, north–south oriented, in Northern East Italy 26 m above sea level (45°35′36″N; 11°56′45″E). The experimental area (45 × 8 m wide) was in a central portion of a greenhouse (2 ha wide) with the span surface divided into four raised beds, 1.6 m wide. Each raised bed was divided in plots of 10 m long, with a surface of 16 m^2^.

The experiment started on 18 October 2022 and ended on 6 March 2023. Two consecutives cultivation cycles using two varieties of baby leaf lettuce (*Lactuca sativa* L.) were evaluated: Doge (Levantia Seed, Rovigo Italy) and Imperiale (Il Mulino, Venice, Italy), which were sowed with a density of 2500 plants m^−2^ in the same raised beds during both cycles. Fertilization treatments were disposed in each plot with two replicates for each treatment. Further details of the timing of the most important agronomic practice during the growing cycles, surveys and measurement activity are provided in Table 1.

**Table 1:** Details of two consecutive crop cycles of baby leaf lettuce, showing the date of fertilization with SPS, date of sowing and the days on which measurements (percentage of covering, SPAD and harvest and post‐harvest surveys) were performed; each date was also transformed into days after sowing (DAS)

Cycle	Fertilization	Sowing	Coverage measurement	SPAD measurement	Harvest and surveys
1st	18 October 2022	22 October 2022	27 October 2022 7 November 2022 23 November 2022	23 November 2022	1 December 2022
DAS			8 19 33	33	40
2nd	14 December 2022	24 December 2022	13 January 2023 2 February 2023 23 February 2023	9 February 2023 23 February 2023	6 March 2023
DAS			21 41 61	47 61	72

SPAD, Soil Plant Analysis Development index.

At the beginning of the experiment, soil samples were taken from the top 20 cm of soil to characterize the initial chemical properties, and the results are shown in Table 2.

**Table 2:** Chemical characterization of soil samples taken at the beginning of the trial at 0–0.2 m of depth

Depth (m)	Organic carbon (%)	Inorganic carbon (%)	Total N	Total P	Total K	Ca	Na	Mg
			(g kg^−1^ DW)					
0–0.20	1.44	0.27	1.67	1.60	6.70	11.58	0.35	7.70

Values are expressed as dry weight (DW).

### Spent pleurotus substrate management

Before each sowing, SPS was collected from the plastic bags at the end of the mushroom cycle. SPS, mainly composed of composted straw and *P. ostreatus* mycelium, was chopped and buried in the soil with a digger and then the soil was levelled and prepared for sowing. Table 3 shows the chemical characterization of SPS used in this study. For each crop cycle, 35 kg ha^−1^ N, 7 kg ha^−1^ P and 4.73 kg ha^−1^ K was used for baby leaf lettuce fertilization. Five fertilization treatments with SPS were tested to satisfy the N crop requirements: T100 + 50 with 100% SPS (12.5 t ha^−1^) to fulfil the N needs of the crop and 50% of additional N through chemical fertilization (Urea, 46%), T200 with 200% SPS (25 t ha^−1^) to fulfil double nitrogen needs of the crop and T200 + 50 with 200% SPS (25 t ha^−1^) to fulfil the double of nitrogen needs of the crop and extra 50% of chemical fertilization (Urea, 46%); T0 was the control without any chemical or organic fertilization and TMIN in which N crop needs (Table 4) were satisfied with only mineral fertilization. The SPS‐containing mineral nitrogen treatments were chosen to limit the immobilization effects due to the high C/N ratio of SPS and make the fertilization treatments more suitable for the short growing cycle of baby leaf lettuce. Phosphorus and potassium nutritional needs were satisfied by SPS distribution in T100 + 50, T200 and T200 + 50 and through mineral fertilization (triple superphosphate [39.1% P_2_O_5_], as well as potassium sulfate [48% K_2_O]) in TMIN. The amounts of SPS and mineral fertilizers used for each treatment are shown in Table 4.

**Table 3:** Chemical properties (expressed as ionic form) and dry matter (%) of Spent Pleurotus Substrate (SPS) used for organic fertilization during all experimental trials. Values are expressed on a dry matter basis

Parameters		SPS
Organic carbon	(%)	31.1
Inorganic carbon	(%)	0.62
Total N	(%)	0.7
C:N ratio		45.3
NO_3_^−^	(mg kg^−1^)	18.1
NO_2_^−^	(mg kg^−1^)	9.90
NH_4_^+^	(mg kg^−1^)	40.5
Total P	(mg kg^−1^)	614
PO_4_^3−^	(mg kg^−1^)	296
K^+^	(g kg^−1^)	259
Mg^2+^	(g kg^−1^)	171
Ca^2+^	(g kg^−1^)	75.9
SO_4_^2−^	(g kg^−1^)	52.4
Na^+^	(g kg^−1^)	39.8
Cl^−^	(g kg^−1^)	157
Dry matter	(%)	36

**Table 4:** Description of fertilization treatments for each crop cycle

N‐P‐K input		T0	TMIN	T100 + 50	T200	T200 + 50
N	(kg ha^−1^)	0	35.0^M^	35SPS + 17.5^M^	70^SPS^	70SPS + 17.5^M^
P	(kg ha^−1^)	0	7.0^M^	80^SPS^	160^SPS^	160^SPS^
K	(kg ha^−1^)	0	4.73^M^	94^SPS^	188^SPS^	188^SPS^

NPK elements are calculated as kg ha^−1^ and derived from organic source (SPS). Mineral source (M) from urea 46% for N, triple superphosphate (39.1%) for P and potassium sulphate (48%) for K.

### Crop surveys

#### Growing phase measurements

During each crop cycle, different assessments were carried out to observe the growth of baby leaf lettuce. With a square frame of 20 × 20 cm, three photos for each plot were taken and analysed with ImageJ (NIH, Bethesda, MD, USA) to obtain the percentage of soil coverage. Then, just before harvest time, leaf chlorophyll index [i.e. Soil Plant Analysis Development index (SPAD) value] was determined with SPAD‐502 Plus (Konica Minolta, Inc., Tokyo, Japan), taking 15 reads for each plot.

#### Harvest measurements

At harvest time, the height of 30 plants was taken. Then, in three sample's area of 900 cm^2^ for each plots, plants’ leaves were cut at 4 cm from soil level, according to the standard practice for the species, and the biomass was measured. Afterwards, the remaining plant parts (crop residues) were also collected and weighed. The former biomass was used to evaluate the commercial yield, whereas the sum of the commercial biomass + crop residues determined the total aerial biomass. A subsample from the commercial biomass was freeze‐dried for qualitative analysis; the remaining parts, along with the non‐commercial parts, were oven dried at 65 °C until constant weight to determine the dry matter content of total and commercial biomass.

Soil samples were randomly collected from the field at the end of each crop cycle using soil sampling scoop that collected about three cores (100 g) from each plot to a depth of approximately 20 cm. All cores were then combined into one composite sample, transported to laboratory and stored at 4 °C before determining DW, active carbon, organic matter and soil enzyme activities (dehydrogenase and total hydrolase activity). Each analysis was carried out on three laboratory replicates.

At the end of each crop cycle, crop residues were burned and then buried in the soil, which was prepared for the next sowing.

### Nitrogen use efficiency

Nitrogen use efficiency was evaluated using the approach suggested by Fageria *et al*.^27^, calculating: agronomic efficiency (AE), physiological efficiency (PE), apparent recovery efficiency (ARE) and utilization efficiency (UE).

Nitrogen indices were calculated using:

$$\mathrm{AE}\;\left(\mathrm{kg}\;{\mathrm{kg}}^{-1}\right)=\mathrm{Gf}-\mathrm{Gu}/\mathrm{Na}$$

$$\mathrm{PE}\;\left(\mathrm{kg}\;{\mathrm{kg}}^{-1}\right)=\mathrm{BYf}-\mathrm{BYu}/\mathrm{Nf}-\mathrm{Nu}$$

$$\mathrm{ARE}\;\left(\%\right)=\left(\mathrm{Nf}-\mathrm{Nu}/\mathrm{Na}\right)\times 100$$

UE=PE×ARE

where *Gf* is the commercial biomass of the fertilized plots (kg), *Gu* is the commercial biomass of the unfertilized plots (kg), *Na* is the quantity of N applied (kg), *BYf* is the total biomass of the fertilized plots (kg), *BYu* is the total biomass of the unfertilized plots (kg), *Nf* is the N uptake (from total biomass) of the fertilized plots and Nu is the N uptake (from total biomass) of the unfertilized plots (kg).

### Qualitative surveys

#### Chemical characterization of soil and SPS

Regarding the determination of the content of anions and cations, ion chromatography (IC) was performed using an Metrohm 930 Compact IC Flex (Metrohm Corp., Herisau, Switzerland) equipped with a dual piston pump, IC autosampler plus 919, isocratic column at controlled temperature (35°C), conductivity detector and chemical suppressor for anions. MagIC Net software (Metrohm Corp.) was used for system control and data processing. A Metrosep A Supp 5‐250/4.0 analytical column and guard column (4 × 50 mm) (Metrohm Corp.) were used for anion separation, whereas a Metrosep C 6‐250/4.0 analytical and guard column (4 × 50 mm) (Metrohm Corp.) were used for cation separation. The eluent consisted of 3.2 mmol L^−1^ sodium carbonate and 1 mmol L^−1^ sodium bicarbonate at a flow rate of 0.7 mL min^−1^ for anions and of 4 mmol L^−1^ nitric acid and 0.7 mmol L^−1^ oxalic acid at a flow rate of 0.9 mL min^−1^ for cations. Anions and cations were quantified following a calibration method. Dionex Seven Anion Standard II (prod. no 057590; Thermo Fisher Scientific, Waltham, MA, USA), Anion multi‐element standard I (product no. 1.11437.0500; Merk, Rahway, NJ, USA), Anion multi‐element standard II (product no. 1.11448.0500; Merk), Lithium Standard for IC (product no. 59878; Sigma‐Aldrich, St Louis, MO, USA), Sodium Standard for IC (product no. 43492; Sigma‐Aldrich), Potassium Standard for IC (product no. 53337; Sigma‐Aldrich), Calcium Standard for IC (product no. 39865; Sigma‐Aldrich), Magnesium Standard for IC (product no. 89441; Sigma‐Aldrich) and Ammonium Standard for IC (product no. 59755; Sigma‐Aldrich) at different concentrations were taken as standards and the calibration curves were generated, with concentrations ranging from 0.1 to 100 mg L^−1^. Nitrogen content in soil samples was measured in accordance with the Kjeldhal method.

Concerning the elements P, and K, the dry samples were mineralized following the method proposed by Zancan *et al*.^28^ Samples were then filtered, and the solution was used for element determination, with a SPECTRO Ciros emission spectrophotometer via inductively coupled plasma–atomic emission spectroscopy (Spectrum Italy Srl, Milan, Italy).

#### Soil samples and analyses

Fresh soil was weighed (8 g) and oven dried at 105 °C until constant weight to determine soil DW. Soil samples were air‐dried and homogenized through a 1‐mm sieve before active carbon determination at 550 nm using an UV‐visible spectrophotometer (DU‐50 UV‐visible; Beckman Instruments, Inc., Fullerton, CA, USA).^29^ Organic matter was assessed by loss on ignition (5 h at 600 °C). The results were expressed as DW.

Dehydrogenase activity (DHA, E.C. 1.1) was assayed using tetrazolium salt as substrate according to Moeskops *et al*.^30^ Total hydrolytic activity (THA) was measured by the fluorescein diacetate method as potential total enzymatic (protease, lipase, non‐specific esterase).^31^ The enzyme activities were expressed as μg^–1^ product g^–1^ DW soil h^–1^.

#### Statistical analysis

The study was arranged as a 5 × 2 × 2 factorial experiment (five fertilization treatments × two varieties × two crop cycles) in a completely randomized block design with three replications. For statistical analysis, RStudio (Posit PBC, Boston, MA, USA) was used to perform a three‐way analysis of variance (ANOVA), for a significant *F*‐value, and means were compared with Tukey's honestly significant difference (HSD) test. *P* < 0.05 was considered statistically significant.

## RESULTS

### Growth and yield measurements

**Figure 1:**
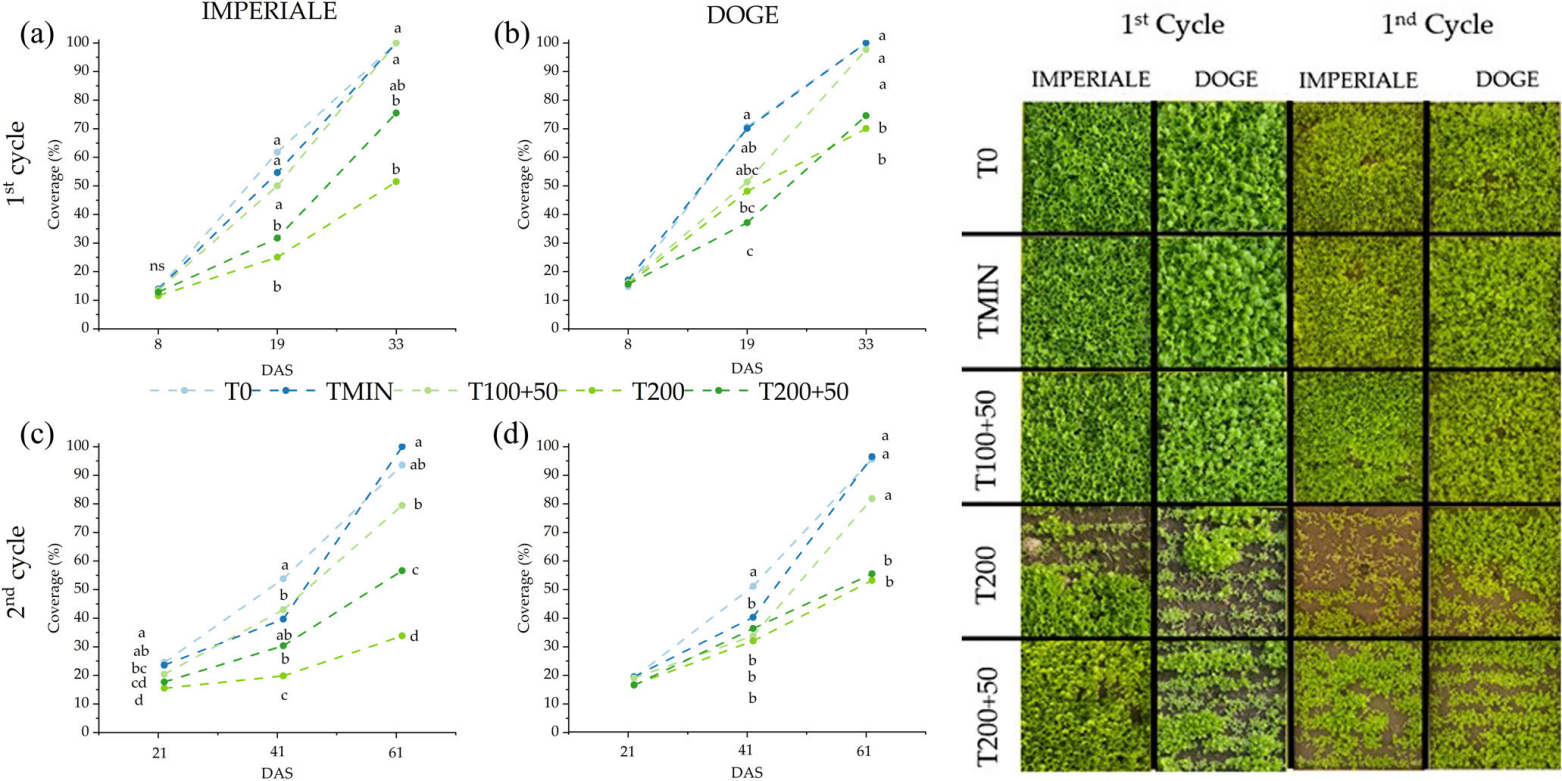
Vegetation coverage (%) of baby leaf lettuce variety Imperiale (a) and Doge (b) in the 1st crop cycle and Imperiale and Doge in the 2nd crop cycle (c, d). Days of measurement are indicated as DAS (days after sowing). Different letters show significant differences with *P* < 0.05 according to Tukey's HSD test. The right image showed photos of vegetation coverage at 33 DAS and 61 DAS in the 1st and 2nd cycles, respectively, for both varieties.

The percentage of vegetation coverage was measured at different times during each crop cycle (Fig. 1). At 19 days after sowing (DAS), both varieties showed a significant difference between treatments. Imperiale had more than 50% of coverage in T0, TMIN and T100 + 50, whereas T200 and T200 + 50 showed a lower growth with a percentage below 30%. Doge variety had the same trend, but for T200 and T200 + 50, the coverage was higher than Imperiale (Fig. 1a,b). At the end of crop cycle (33 DAS), T0, TMIN and T100 + 50 obtained 100% of coverage in both varieties, whereas T200 + 50 and T200 treatments obtained less than 80% of coverage. In the 2nd cycle of cultivation (Fig. 1c,d), after 21 days, Imperiale showed significant differences between treatments with T0 (24.53%) higher than T200 (15.47%). During the second surveys (41 DAS), both Imperiale and Doge showed significant differences according to the treatment: Imperiale had the higher percentage in T0 and T100 + 50 treatments (53.77% and 42.95%, respectively) whereas Doge obtained the higher coverage in T0 (51.19%). At harvest, TMIN reached 100% of coverage for Imperiale, whereas, for Doge T0, TMIN and T100 + 50 obtained the higher coverage compared to T200 and T200 + 50, which were below 60%.

**Figure 2:**
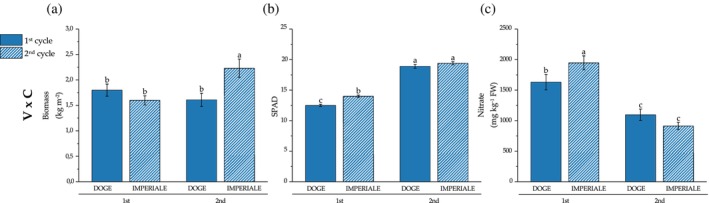
Influences of two‐way interactions of variety and crop cycle (V × C) of plant biomass (a) expressed as kg m^−2^ FW, SPAD index (b) and nitrate content mg kg^−1^ FW (c). Different letters show significant differences with *P* < 0.05 according to Tukey's HSD test.

Table 5 shows fresh total biomass, SPAD index and nitrate content of each crop cycle. Plant total biomass production was significant for all three main factors: fertilization treatment (FT), variety (V) and crop cycle (C). Imperiale produced a higher biomass compared to Doge. Also, the 2nd cycle had higher biomass production compared to the 1st one. Fertilization treatments were highly significant (*P* < 0.001) and T0 and TMIN had the higher value of biomass (on average, 2.46 kg m^−2^), T100 + 50 produced an average of 1.99 kg m^−2^ (−24.0% compared to T0). At last, T200 and T200 + 50 had the lowest biomass value, on average −54.8% compared to T0. Also, the interaction shown in Fig. 2(a) between crop cycle and variety showed a higher significance (*P* < 0.001) with Imperiale × 2nd cycle which had a higher biomass value compared to Imperiale × 1st cycle (−39.4%); on the other hand, Doge obtained the lower biomass value with 1.80 and 1.61 kg m^−2^ in the 1st and 2nd cycle, respectively.

**Table 5:** Biomass (kg m^−2^ FW), SPAD index and nitrate content (mg kg^−1^ FW) measured at the harvest of the 1st and 2nd crop cycle of two varieties of baby leaf lettuce: Doge (D) and Imperiale (I)

	Biomass (kg m^−2^ FW)		SPAD		Nitrate (mg kg^−1^ FW)	
Fertilization treatment (FT)							
T0	2.6	± 0.13 a	18.2	± 0.5 a	1127	± 148
TMIN	2.3	± 0.09 ab	16.5	± 0.4 b	1444	± 159
T100 + 50	2.0	± 0.15 b	15.7	± 0.4 b	1369	± 137
T200	1.2	± 0.09 c	15.7	± 0.4 b	1486	± 129
T200 + 50	1.2	± 0.07 c	16.1	± 0.3 b	1391	± 120
Significance	***		***		NS		
Variety (V)							
Doge (D)	1.7	± 0.09 b	15.9	± 0.3 b	1349	± 84.2
Imperiale (I)	1.9	± 0.11 a	16.8	± 0.2 a	1403	± 92.5
Significance	*		***		NS		
Cycle (C)							
1st	1.7	± 0.08 b	13.3	± 0.1 b	1789	± 86.3 a
2nd	1.9	± 0.12 a	19.1	± 0.2 a	1005	± 56.4 b
Significance	*		***		***		

Means are followed by the SE; different lowercase letters show significant differences between treatments according to Tukey's HSD test.

*Note*: NS, not significant.

* *P* < 0.05.

*** *P* < 0.001.

**Figure 3:**
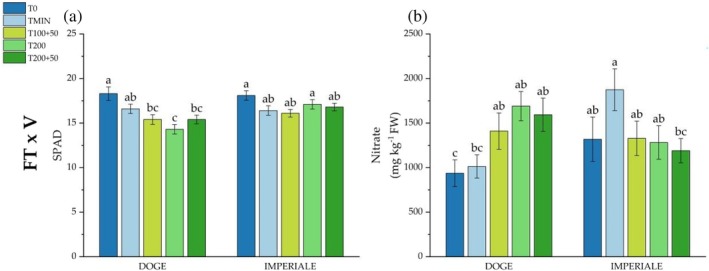
Influences of two‐way interactions of fertilization treatment and variety (FT × V) on SPAD index (a) and nitrate content (mg kg^−1^ FW) (b). Different letters show significant differences with *P* < 0.05 according to Tukey's HSD test.

Regarding leaf SPAD value, both fertilization treatments, variety and crop cycle showed significant effects (Table 5). Fertilization treatments showed the higher SPAD value in T0 (18.2) compared to all the other treatments (16.0, on average). Imperiale showed a higher SPAD values than Doge, whereas the crop cycle has a significant effect on this variable, with a higher index in the 2nd crop cycle compared to the 1st one. In the interaction ‘FT × V’ shown in Fig. 3(b), SPAD values in Imperiale were similar among fertilization treatments, whereas, in Doge, some differences were observed, with T0 showing the higher value and T100 + 50, T200 and T200 + 50 showing the lower value. Also, the interaction ‘V × C’ showed that, in the 1st cycle, Imperial had higher SPAD value than Doge, whereas, in the 2nd cycle, no difference was observed (Fig. 2(b)).

Nitrate content in the commercial product was not affected both by fertilization treatments and varieties. Crop cycle was statistically significant for nitrate content, with the 1st having an average value of 1789 mg NO^−3^ kg^−1^ fresh weight (FW), which was 78.0% higher compared to the 2nd cycle. The interaction ‘FT × V’ was significant (Fig. 3b) Imperiale had higher nitrates content in T0 and TMIN (1318 and 1874 mg NO^−3^ kg^−1^ FW, respectively), whereas Doge, with the same fertilization treatments, accumulated the lowest concentrations (937 and 1013 mg NO^−3^ kg^−1^ FW, respectively). In Fig. 2(c), the ‘V × C’ interaction showed that, in the 1st cycle, Doge's nitrate content was higher compared to Imperiale, whereas, in the following cycle nitrate content was the same for both varieties (in the range 1097–913 mg NO^−3^ kg^−1^ FW).

### Nitrogen indices and balance

Table 6 shows the results of ANOVA analysis for the different nitrogen efficiency indices. Agronomic efficiency (AE) showed highly significant differences for all main factors and interactions. For fertilization treatments T200 + 50 and T100 + 50 obtained the higher values (−62 kg kg^−1^ N and – 74.8 kg kg^−1^ N) and T200 obtained the lowest (−97.9 kg kg^−1^ N). Regarding the other main factors, a higher value was found in the 1st cycle (+53.5% compared to the 2nd cycle) and also for Imperiale variety (+20.8% compared to Doge).

**Table 6:** Agronomic efficiency (AE), physiological efficiency (PE), apparent recovery efficiency (ARE) and utilization efficiency (UE) of 1st and 2nd cycle of Doge and Imperiale varieties

	AE (kg kg^−1^)		PE (kg kg^−1^)		ARE (%)		UE	
Fertilization treatments (FT)								
TMIN	−91.4	± 12.3 bc	−5.53	± 112	−4.99	± 5.10	−460.0	± 1338 a
T100 + 50	−74.8	± 97.9 ab	254	± 296	−12.2	± 4.08	−4388	± 1236 ab
T200	−97.9	± 6.39 c	815	± 337	−17.8	± 4.67	−7460	± 1821 b
T200 + 50	−62.0	± 5.82 a	466	± 27.8	−15.5	± 3.65	−5767	± 1378 b
Significance	***		NS		NS		***	
Variety (V)								
Doge (D)	−89.2	± 5.25 b	509	± 183	−10.1	± 3.22	−4812	± 8364
Imperiale (I)	−73.8	± 7.74 a	255	± 149	−15.2	± 3.06	−4226	± 6613
Significance	***		NS		NS		NS	
Cycles (C)								
1st	−51.6	± 5.31 a	504	± 210	−24.9	± 3.70 b	−8874	± 1251 b
2nd	−111	± 4.85 b	260	± 107	−0.32	± 0.04 a	−164.0	± 17.90 a
Significance	***		NS		***		***	

Different lowercase letters indicate significant differences between treatments with *P* < 0.05 according to Tukey's HSD test.

Note: NS, not significant.

*** *P* < 0.001.

**Figure 4:**
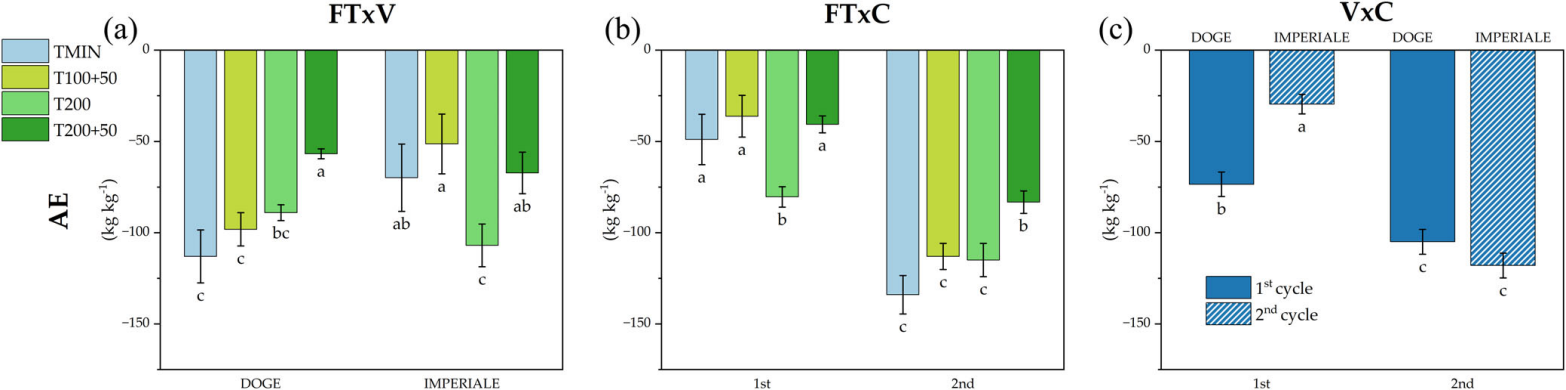
Influences on agronomical efficiency (AE) of two‐way interactions of fertilization treatment and variety (FT × V) (a), fertilization treatment and crop cycle (FT × C) (b) and variety and crop cycle (V × C) (c). Different letters show significant differences with *P* < 0.05 according to Tukey's HSD test.

For the interaction ‘FT × V’ shown in Fig. 4(a), Imperiale obtained an higher AE value in TMIN, T100 + 50 and T200 + 50 compared to Doge, which had an higher value only in T200 + 50. Regarding of ‘FT × C’ interaction (Fig. 4(b)) in the 1st cycle, T200 outperformed the other trestments, whereas, in the 2nd cycle, higher values were obtained by T200 + 50. Lastly, the interaction ‘V × C’ showed that Imperiale had an higher AE in the 1st cycle compared to Doge, but, in the 2nd crop cycle, values of AE index were not statistically different between varieties (Fig. 4c). Although PE was not affected by main factors and interactions, ARE was only affected by crop cycle: higher values were found in the 2nd (−0.32%) compared to the 1st cycle (−24.9%) (Table 6). EU index showed significant differences for fertilization treatments: TMIN (−460 kg kg^−1^) had higher values compared to T200 and T200 + 50 (−7460 kg kg^−1^ and −5767 kg kg^−1^), as well as higher values in the 2nd cycle compared to the 1st. Also for EU the interaction “FT × C was significant (*P* < 0.001) with TMIN reporting the highest values in the 1st cycle compared to the other treatments, whereas no difference was observed in the 2nd crop cycle.

**Figure 5:**
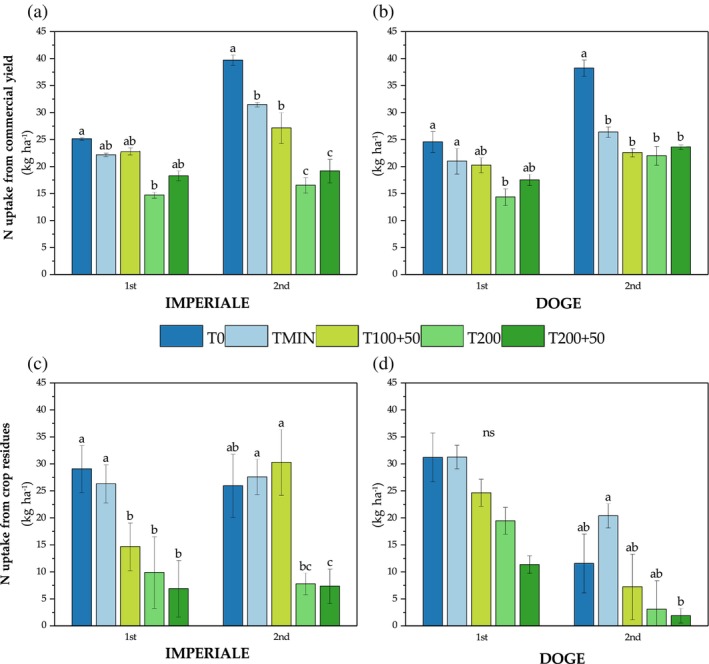
Influences on agronomical efficiency (AE) of two‐way interactions of fertilization treatment and variety (FT × V) (a), fertilization treatment and crop cycle (FT × C) (b) and variety and crop cycle (V × C) (c). Different letters show significant differences with *P* < 0.05 according to Tukey's HSD test.

Figure 5(a) shows the nitrogen uptake from the commercial yield in both crop cycle for Imperiale's variety where T0 had the highest uptake (25.1 kg ha^−1^) and T200 the lowest (14.7 kg ha^−1^). In the 2nd crop cycle, T0 uptake was again the highest (39.7 kg ha^−1^) and T200 was the lowest, but similar to that of T200 + 50 (17.1 kg ha^−1^); TMIN and T100 + 50 had a similar intermediate uptake (29.3 kg ha^−1^, on average).

Doge variety showed different N uptake from commercial yield (Fig. 5b). T0 had the same result of TMIN in the 1st cycle with 24.6 and 21.0 kg ha^−1^ respectively; T200 was the treatment with the lower uptake compared to T0 (−41.2%). In the 2nd cycle, N uptake was higher in T0 compared to all the other treatments with no other difference among them, reaching 38.3 kg N ha^−1^. Nitrogen uptake from crop residues for Doge (Fig. 5d) was not statistically different in the 1st crop cycle, whereas, in the following cycle, TMIN had the highest uptake (20.4 kg ha^−1^), whereas T200 + 50 had the lowest (1.9 kg ha^−1^). N uptake from residues for Imperiale, shown in Fig. 5(c), was higher in T0 and TMIN compared to all SPS treatments, which were below 15 kg ha^−1^, but, in the 2nd cycle, T100 + 50 and TMIN obtained the higher amount of N in crop residues compared to T200 and T200 + 50 (7.79 and 7.37 kg ha^−1^, respectively).

**Figure 6:**
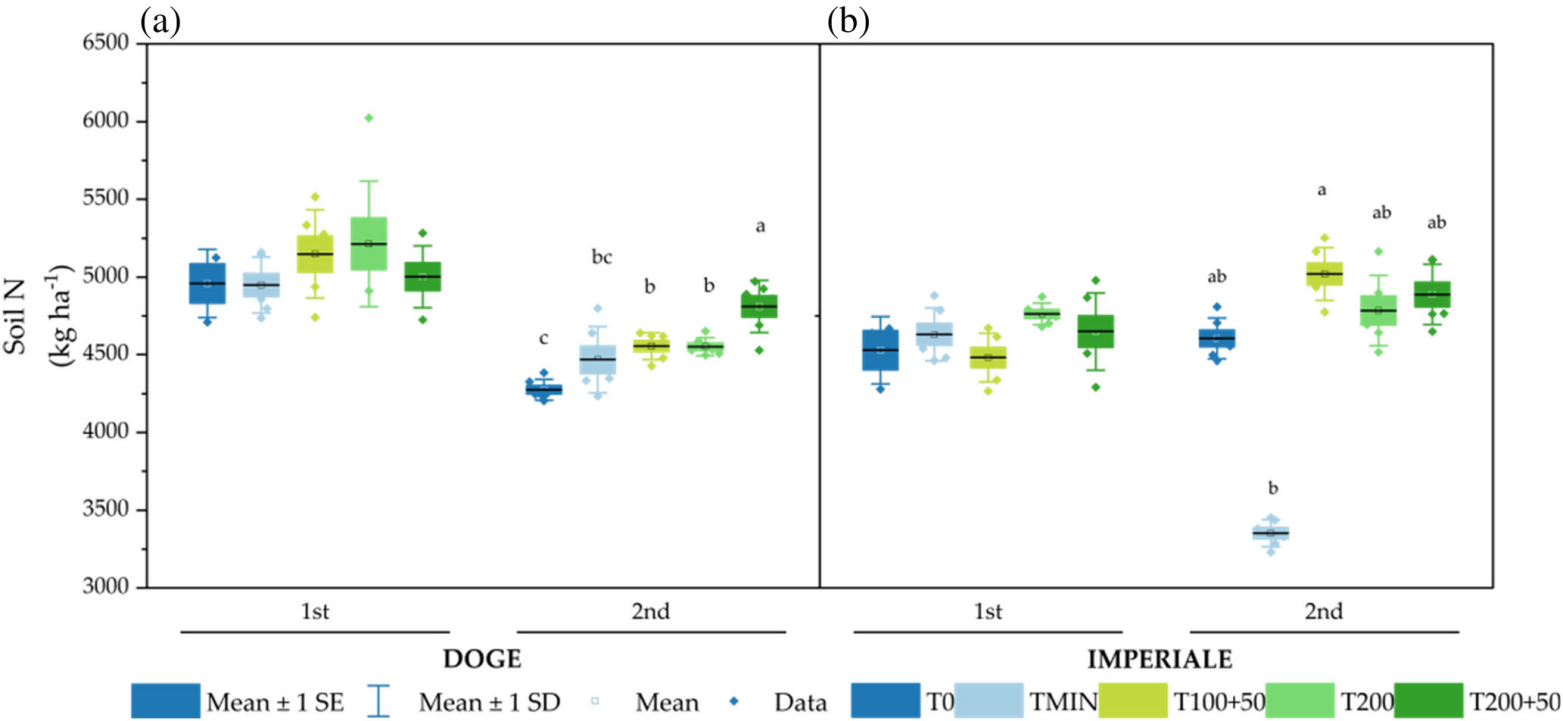
Influences of two‐way interactions of fertilization treatments and crop cycle (FT × C) on N uptake from commercial yield crops for baby lettuce varieties Imperiale (a) and Doge (b) and N uptake from residues of crops for Imperiale (c) and Doge (d) varieties. Different letters indicate significant differences between treatments with *P* < 0.05 according to Tukey's HSD test.

Figure 6(a,b) shows the soil nitrogen content in the 0–20 cm soil depth at the end of every crop cycle. At the beginning of the experiment, the amount of nitrogen in the soil was 4589 kg N ha^−1^ (data not reported). In the 1st cycle, which lasted only 2 months, N present in the soil was not affected by soil treatments for both lettuce varieties, but some statistically differences were observed in the interaction ‘Varieties × Fertilization treatment’ at the end of the 2nd crop cycle. The amount of soil's N in Doge was higher in T200 + 50 compared to all other fertilization treatments (+12.5%, +5,62% and +5.71% in comparison to T0, T100 + 50 and T200 respectively). For Imperiale soil's N content showed a different trend, with a statistical difference only between TMIN and T100 + 50.

### Soil quality

The effects of fertilization treatments and crop cycle were statistically significant for Organic Matter (OM), Active Carbon (Act C), DHA, and THA (showing S.1). T200 was the fertilization treatment with the highest content of OM, Act C and DHA, compared to T0 and TMIN, which consistently showed the lowest values. Regarding the crop cycle, the 2nd cycle had higher values of OM, Act C and DHA compared to the 1st cycle. THA shown in SM1, was statistically significant only for the fertilization treatment, with all SPS treatments exhibiting higher values compared to T0 and TMIN.

**Figure 7:**
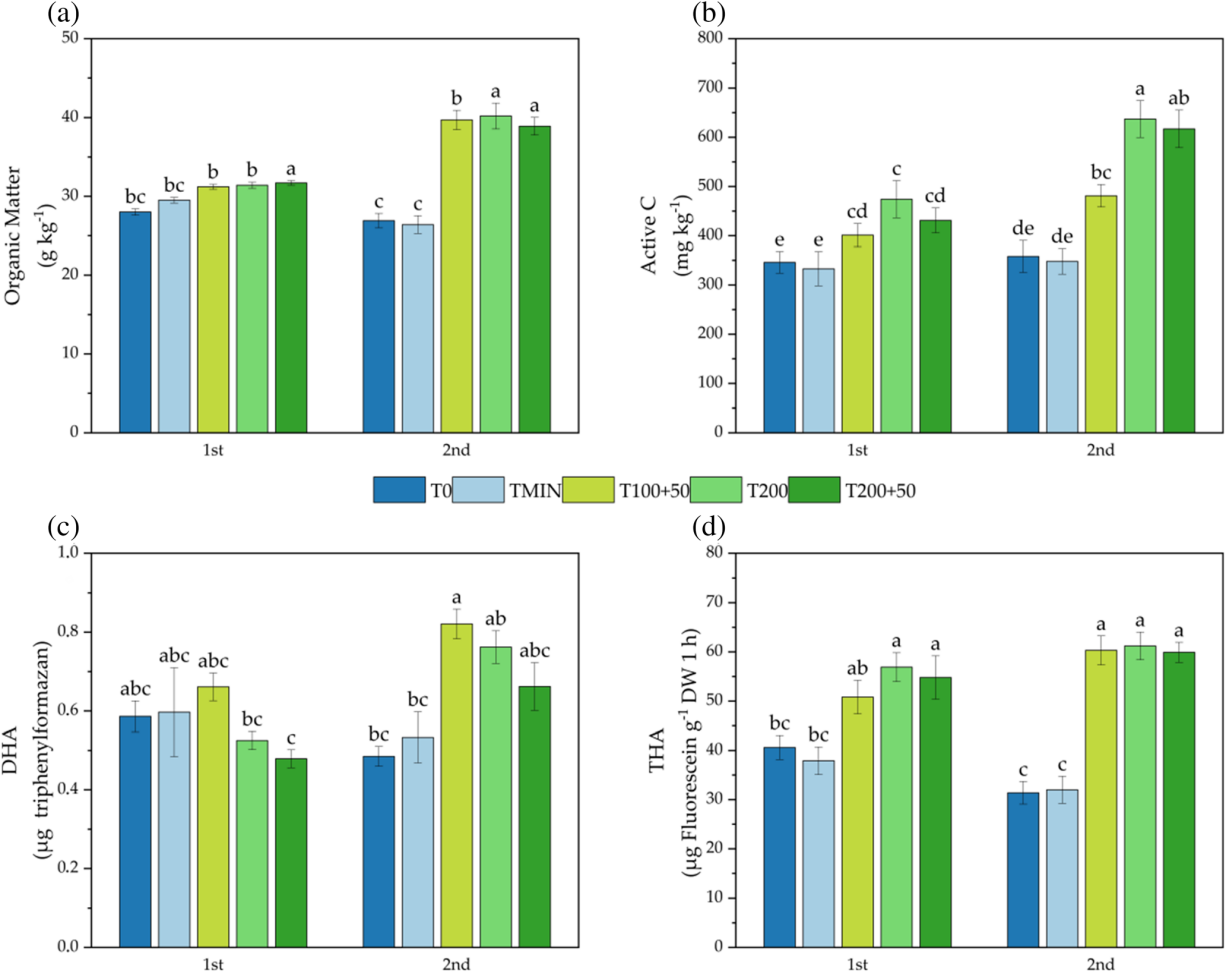
Soil N amount (kg ha^−1^) in both crop cycles for Doge (a) and Imperiale (b) lettuce varieties. Different letters indicate significant differences between treatments with *P* < 0.05 according to Tukey's HSD test.

For OM content, two significant interactions were observed: ‘FT × C’ (Fig. 7a) and ‘V × C’ (S.2). In the ‘FT × C’ interaction, all SPS treatments increased OM content between the 1st and 2nd cycles by +27.2% for T100 + 50, +28.0% for T200 and + 22.7% for T200 + 50, whereas, in T0 and TMIN, OM decreased by −3.93% and − 10.51%, respectively. In the ‘V × C’ interaction, the Doge variety in the 2nd cycle exhibited a higher OM content (35.8 g kg^−1^) compared to the Imperiale variety in the same cycle (33.0 g kg^−1^).

The amount of active carbon also showed significant differences in the ‘FT × C’ interaction (Fig. 7b), with an increase of +34.3% from T200 × 1st to T200 × 2nd, and an increase of +43.1% from T200 + 50 × 1st to T200 + 50 × 2nd. Furthermore, the ‘V × C’ interaction demonstrated a higher amount of Act C in Imperiale × 1st compared to Doge × 1st (SM2).

Finally the enzymes activity of DHA and THA showed significant differences in ‘FT × C’ interaction (as shown in Fig. 7c,d). T100 + 50 × 2nd was higher than T200 + 50 × 1st for DHA, whereas all fertilization treatments with SPS in both cycles showed higher THA activity than T0 and TMIN.

## DISCUSSION

### Growth and yield measurements

Raw SPS application for two consecutive crop cycles of baby leaf lettuce significantly affected growth and crop's productivity. In both cycles and varieties, T0 exhibited the higher value of crop coverage and total biomass. The amount of total N present in the soil (1.67 g kg^−1^) at the beginning of this experiment can be considered high^32^ because of the previous company's fertilization management. In addition, significant variations in soil nitrogen content in control treatments need relevant amount of time to be highlighted. Among SPS treatments T100 + 50 yielded the best growth compared to other SPS treatments: adding 50% of mineral fertilization helped the decomposition rate of the cereal straw as also indicated by Guan *et al*.^33^ who observed a faster mineralization of straw mixed with mineral fertilizer. In this case, the immobilization effect of T100 + 50 did not adversely affect crop productivity. On the other hand, T200 and T200 + 50 decreased crop yield because of a higher nitrogen immobilization as a result of SPS overload. Adding 17.5 kg N ha^−1^ did not improve the decomposition rate in T200 + 50 as it did for T100 + 50, probably because it was not sufficient to counteract for the higher amount of undecomposed organic matter. Different results were observed in the study by Xu *et al*.^34^ where increasing amounts of 1 year‐composted SPS promoted higher yield for lettuce cultivated in a protected environment thanks to the different chemical properties after composting process. In addition, comparing SMS performance is challenging because of the high variability in mushroom substrates. Most studies use SMS from *A. bisporus* as raw or composted,^6^, ^14^, ^18^, ^34^, ^35^, ^36^, ^37^, ^38^ whereas others have used SPS only after a composting process that lasted several months.^6^, ^38^, ^39^, ^40^ These substrate managements can deeply modify all the characteristics of the substrate at the end of mushroom production. The SPS material used in this experiment was raw and has not undergone any previous treatment that altered its composition. This choice was made to see the chemical and biological effects on the soil of the raw material at the end of the mushroom production process and to limit the management costs of this organic by‐product. At the same time, SPS cannot be considered as raw wheat straw because it was degraded by *P. ostreatus* during mushrooms production which changed the number of lignocellulosic components and C/N ratio compared to raw wheat straw (from 100 to 45). Several studies have shown that agricultural residues are degraded by the mycelium of *P. ostreatus* when comparing the cultivation substrate before and after mushrooms production. Adamovic *et al*.^41^ observed a degradation rate of 4% for lignin, 17% for hemicellulose and 15% for cellulose in a cultivation substrate for *P. ostreatus* composed mainly by wheat straw and measured a faster degradation for hemicellulose and lignin. Also, van Kuijk *et al*.^42^ noticed a similar degradation of the same lignocellulosic compounds. So, after a production cycle of mushrooms, part of straw's lignin and hemicellulose is already degraded and transformed into glucose and xylose and could be more easily degraded by microorganisms. Furthermore, the presence of extracellular enzymes belonging to lignocellulolytic group is useful to make straw degradation faster as also indicated by Guan *et al*.^33^ Usually, for straw incorporation in open field, the mineralization rate is faster in the early phase in a summer–autumn cycle and in the last stage in a winter–spring cycle.^43^ Hence, the season in which our experiment was carried out surely affected the speed of SPS degradation. The 1st and the 2nd crop cycle occurred in the autumn–winter season and, even though the trials took place in a greenhouse, the temperature was not high enough to aid SPS mineralization except in the early stage of the 1st cycle and the last month of the 2nd cycle. Varieties exhibited similar growth behaviour with SPS treatments, but Imperiale outperformed Doge, especially in the 2nd cycle. Overall, the 2nd cycle had higher growth as a result of increasing seasonal photoperiods affecting baby leaf biomass and Spad index.

Imperiale and Doge showed a different behaviour in nitrogen uptake from organic and mineral sources. Nitrate uptake is an active transport which requires energy and carbohydrates, leading to differences in nitrate uptake among different lettuce varieties if they have different photosynthetic capacities.^44^ In the present study, the SPAD index was different among varieties. Lettuce's crop mainly accumulates nitrate in the leaf but crop can also store it in the roots during an excess absorption. This behaviour could explain the low amount of nitrate found in the commercial samples of Doge variety in TMIN.^45^ Nevertheless, SPS treatments did not cause a higher nitrate content in the product, which was below the maximum amount indicated by Reg. UE 1258/2011. On the other hand, only the 1st crop cycle affected the nitrate content with a higher value (harvest in December) compared to the 2nd cycle (harvest in March) because of a longer photoperiod and a higher radiation intensity which decrease nitrate accumulation.^46^

### Nitrogen use efficiency

Nitrogen use efficiency indices revealed few significant differences between treatments, likely as a result of the short crop cycles and the cold season, which affect organic matter mineralization and nutrient dynamics. The AE index results were negative, indicating high soil fertility in T0 compared to all other fertilization treatments and a lower efficiency of the nitrogen supplied trough the fertilization. TMIN and T200 showed the lowest AE results, demonstrating that mineral fertilization and SPS treatments alone may be less efficient compared to treatments that combine organic and mineral fertilization. This combination, as seen in T100 + 50 and T200 + 50, helped the mineralization of SPS and increased nutrient availability, as suggested by Liu *et al*.^47^ and Guan *et al*.^33^ Additionally, Nicoletto *et al*.^48^ found reduced AE indices in two different lettuce species when higher quantities of anaerobic digestion residues were used. However, in this case, the C/N ratio of SPS is higher than that of anaerobic digestion residues, making a direct comparison between the two organic fertilizers inappropriate. The AE index showed statistically significant differences for all factors and interactions: Imperiale demonstrated a better ability than Doge to utilize nitrogen in fertilization treatments involving mineral fertilization (TMIN, T100 + 50, and T200 + 50). Similarly, Santamaria *et al*.^49^ observed differences in nitrogen use efficiency between two rocket salad ecotypes and Di Gioia *et al*.^50^ reported variations in AE index between two lettuce species with increasing nitrogen fertilization rates. In the present study, lettuce varieties significantly affected several productive parameters and nitrogen uptake and use. These effects were discussed in relation to soil quality parameters such as DHA and THA, suggesting a correlation between different enzymatic activities in the soil and nitrogen uptake by the various lettuce varieties.

At the same time, the growing cycle of SPS treatment application affected nitrogen fertilization efficiency: TMIN, T100 + 50 and T200 + 50 showed a better efficiency in the 1st crop cycle compared to the 2nd one that occurred in wintertime (from December to March); thus, indicating that the accumulation of two successive SPS fertilization treatments decreased nitrogen use efficiency overall.

EU index did not show analogous result with higher values in TMIN and T100 + 50 caused by its correlation with the total biomass and not with commercial yield. Crop cycles had significant differences for AE, ARE and EU indices. AE demonstrated as in the 1st crop cycle fertilization treatments were more effective for the yield compared to the 2nd, whereas ARE and UE, calculated considering the amount of *N* uptake by the crops, had better results in the 2nd crop cycle. These results could be explained with the succession of fertilization treatments applied in the soil in a short period of time in addition to the different light and temperature condition during the last part of the cycle which did not help the mineralization of SPS treatments in the soil. Additionally, in the 2nd crop cycle, the longer photoperiod and the higher temperature occurred when plant had reached a high growth stage, resulting in higher total photosynthate accumulation, available for nitrogen uptake.

Nitrogen uptake from commercial biomass was higher in T0, TMIN for the easier uptake of the crop from the mineral source present in the soil, in T100 + 50 and T200 + 50 in the 1st cycle where the amount of mineral nitrogen applied to the soil was available for the crop and helped microorganisms to mineralize SPS. Nitrogen uptake for SPS was lower in the 2nd cycle because low temperature slowed down microorganism activity making nitrogen less available for the crops. Nitrogen uptake from crop residues is crucial for its subsequent incorporation into the soil that can further increase N level into the soil. TMIN and T0 had, for both varieties, higher N uptake, and T100 + 50 for Imperiale.

The nitrogen content in the top 20 cm of soil was enhanced by SPS in just two crop cycles (6 months), with the Imperiale variety and SPS treatments showing the highest values, indicating an increase in nitrogen availability for the next crop. A similar trend was observed with the Doge variety, although SPS did not enhance nitrogen levels as much as with Imperiale, maintaining a consistent level instead. Additionally, the increased nitrogen content in the soil could benefit subsequent crop cycles because the mineralization of previous fertilization treatments may help microorganisms counteract the immobilization effect of new SPS incorporation.

### Organic matter, active carbon and enzyme activities

The application of SPS increased, as expected, the OM content compared to mineral fertilization and control treatment. After 2 months from SPS application, at the end of the 1st cycle, T200 + 50 showed a higher amount of OM compared to TMIN; at the end of the 2nd cycle (6 months after the beginning of the experiment) all SPS treatments showed a significant OM increase. This result is in agreement with that reported previously in studies using spent substrate from *Agaricus bisporus*, *Auricularia auricula* and Enoki mushroom.^14^, ^51^, ^52^ The OM in the soil was even influenced by the lettuce variety as demonstrated in the 2nd cycle. This trend could be explained by the differences noticed within the microbial community in the root zone and the different enzyme activity of DHA. The latter relates to the OM content in the soil and was higher in Doge compared to Imperiale. The content of Act C was higher in the SPS treatments with the higher doses (T200 and T200 + 50) and in the 2nd cycle caused by the accumulation of SPS after 6 months of experiment. A different behaviour of the two variety of baby leaf lettuce was observed on DHA enzyme activity and Act C, this result can be explained by a different relationship between baby lettuce variety microorganisms and nitrogen uptake: Doge was able to absorb more nitrates from SPS thanks to the higher activity of DHA, whereas Imperiale up took more nitrate from mineral source and had less DHA but more Act C an easy food source for microorganisms.

## CONCLUSIONS

The application of raw spent *Pleurotus* substrate (SPS) over two consecutive crop cycles of baby leaf lettuce demonstrated significant effects on growth and productivity, with notable differences observed between treatments and varieties. The control treatment (T0) consistently produced higher crop coverage and total biomass compared to SPS treatments, highlighting the importance of effective fertilization strategies.

Among the SPS treatments, T100 + 50 yielded the best results in terms of production, suggesting that the combination of 50% mineral fertilization with SPS enhanced decomposition rates, thereby improving nutrient availability without negatively impacting crop yield. Conversely, higher SPS concentrations (T200 and T200 + 50) led to decreased yields due to excessive nitrogen immobilization.

The present study revealed that the nitrogen content in the soil improved with SPS application, particularly with the Imperiale variety, which exhibited superior nitrogen uptake and utilization compared to Doge. The timing and seasonal conditions of the crop cycles also played a significant role in nitrogen dynamics and crop performance, with the 1st cycle showing better efficiency in mineralization and the 2nd cycle higher yield. Furthermore, SPS significantly increased organic matter, active carbon and enzyme activity in the soil, contributing to enhanced microbial activity and nutrient cycling. The differing behaviors of the lettuce varieties in terms of nitrogen uptake and enzyme activity suggest that variety selection is critical for optimizing the benefits of SPS application. The findings underscore the potential of raw SPS as a valuable organic fertilizer, particularly when combined with mineral fertilization, to improve soil fertility and crop productivity in sustainable agricultural practices. Future research should explore long‐term effects and further optimize SPS application rates and combinations to maximize benefits across different baby leaf species and growing conditions.

## AUTHOR CONTRIBUTIONS

MDB and CN were responsible for conceptualization. MDB and CN were responsible for methodology. MDB and CN were responsible for validation. MDB, MC and CN were responsible for formal analysis. MDB, MC and CN were responsible for investigation. CN and PS were responsible for resources. MDB, MC and CN were responsible for data curation. MDB was responsible for writing the original draft. MDB, MC, GZ and CN were responsible for reviewing and editing. MDB, MC and CN were responsible for visualization. PS, GZ and CN were responsible for supervision. CN was responsible for project administration. CN was responsible for funding acquisition. All authors have read and approved the final version of the manuscript submitted for publication.

## FUNDING

This research was funded by FSE REACT‐EU, PON Ricerca e Innovazione 2014–2020 Action IV.5 – GREEN and Società Agricola Mancon (https://www.mancon-sa.it; accessed 30 March 2024).

## Supporting information


**Data S1.** Supporting Information.

## Data Availability

The data that support the findings of this study are available from the corresponding author upon reasonable request.
