# Supplementary material for: Spent Pleurotus substrate as organic fertilizer to improve yield and soil fertility: the case of baby leaf lettuce production
Source: J Sci Food Agric. 2025 May 28;105(11):5874–86. doi: 10.1002/jsfa.14303 (PMC12260337; doi:10.1002/jsfa.14303)

Supplementary materials:

*S.1 – Effect of Soil treatments (T0, TMIN, T100+50, T200 and T200+50), baby leaf lettuce varieties (Imperiale and Doge) and Cycle (1^st^ and 2^nd^) on soil organic matter (SOM), active carbon soil (Act C), Dehydrogenase activity (DHA) (µg triphenylformazan) and Total Hydrogenase Activity (THA) (µg Fluorescein g^-1^ DW 1h). Means are followed by standard error; different letters indicate significant differences between treatments with p-value<0.05 according to HSD Tukey Test.*

|  | OM | | Act C | | DHA | | THA | |
| --- | --- | --- | --- | --- | --- | --- | --- | --- |
|  | g kg-1 | | mg kg-1 | | µg triphenylformazan | | µg fluorescein g^-1^ DW 1h | |
| Fertilization Treatments (FT) | | | | |  | | | |
| T0 | 27.4 | ±0.490 **b** | 351 | ±18.1 **c** | 0.546 | ±0.027 **b** | 36.9 | ±1.90 **b** |
| TMIN | 28.2 | ±0.568 **b** | 339 | ±22.8 **c** | 0.571 | ±0.072 **b** | 35.5 | ±2.06 **b** |
| T100+50 | 35.0 | ±1.001 **a** | 433 | ±18.1 **b** | 0.725 | ±0.029 **a** | 54.6 | ±2.48 **a** |
| T200 | 34.9 | ±1.055 **a** | 539 | ±30.5 **a** | 0.620 | ±0.030 **ab** | 58.6 | ±2.10 **a** |
| T200+50 | 34.9 | ±0.874 **a** | 505 | ±27.0 **ab** | 0.552 | ±0.032 **b** | 56.8 | ±2.78 **a** |
| Variety (V) | | | | |  | | | |
| Doge | 32.5 | ±0.750 | 398 | ±17.2 **b** | 0.640 | ±0.033 **a** | 47.7 | ±1.94 |
| Imperiale | 31.7 | ±0.571 | 470 | ±17.0 **a** | 0.566 | ±0.019 **b** | 49.3 | ±1.76 |
| Cycle (C) | | | | |  | | | |
| 1^st^ | 30.4 | ±0.221 **b** | 397 | ±13.9 **b** | 0.570 | ±0.026 **b** | 48.2 | ±1.64 |
| 2^nd^ | 34.4 | ±0.989 **a** | 488 | ±21.0 **a** | 0.653 | ±0.027 **a** | 49.0 | ±2.16 |

*S.2: Significant interaction “C × V” of Organic Matter (g kg^-1^) (S.2a) and Active Carbon (mg kg^-1^) (S.2b). Different letters indicate significant differences between treatments with p-value<0.05 according to HSD Tukey Test.*


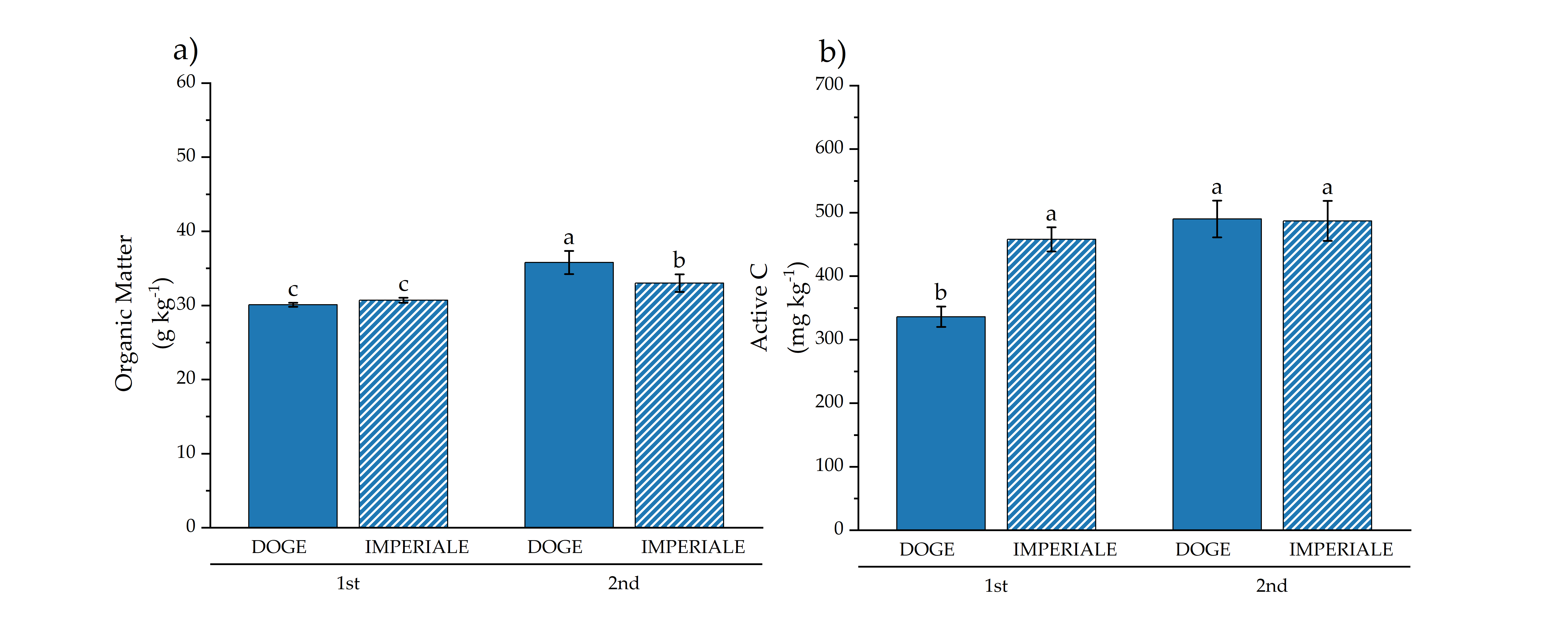

Supplement: Supplementary file 1 — Data S1. Supporting Information. [file JSFA-105-5874-s001.docx]
